# Understanding Barriers and Facilitators to Signing Up for a Mobile-Responsive Registry to Recruit Healthy Volunteers and Members of Underrepresented Communities for Alzheimer’s Disease Prevention Studies

**DOI:** 10.14283/jpad.2023.67

**Published:** 2023-06-06

**Authors:** D. Neffa-Creech, R. Aggarwal, C. Stowell, U. Menon, S. George, A. Plant, Jessica Langbaum

**Affiliations:** 1 Sentient Research, West Covina, CA USA; 2 Provoc, Washington, District of Columbia, USA; 3 University of South Florida Health College of Nursing, Tampa, FL USA; 4 Department of Preventive and Social Medicine, Charles R. Drew University of Medicine and Science and Department of Community Health Sciences, UCLA, Los Angeles, CA USA; 5 Banner Alzheimer’s Institute, 901 E. Willetta Street, Phoenix, AZ 85006 USA

**Keywords:** Alzheimer’s disease, underrepresented communities, participant recruitment registry, mixed methods, prevention

## Abstract

**Background:**

Alzheimer’s disease (AD) disproportionately affects Black/African American and Hispanic/Latino adults, yet they are underrepresented in AD studies. Recruitment challenges for these populations limit generalizability of findings.

**Objectives:**

This study explores barriers and facilitators to signing up for an AD participant recruitment registry website intended to optimize recruitment of these adults. The registry is geared toward recruitment on smartphones and tablets (mobile devices), as research suggests that mobile-first approaches may be more successful within these populations.

**Design:**

In 2020, we conducted four focus groups (n = 39) and an online survey (n = 1010) with Black/African American and Hispanic/Latino adults. The survey also included Whites as a comparison group.

**Setting:**

Focus groups were in-person at research facilities in New Orleans, Louisiana, and Los Angeles, California. The online survey was distributed by a survey panel company to participants nationwide.

**Participants:**

Black/African American (n = 360), Hispanic/ Latino (n = 359), or White (n = 330) individuals, 45–75 years old, who self-reported not having mild cognitive impairment (MCI), dementia, or AD.

**Measurements:**

Barriers and facilitators explored in the focus groups and survey were related to health and AD (e.g., AD-related concerns and past participation/willingness to participate in health or AD studies); current use of mobile devices (e.g., comfort using devices and receptivity to the AD recruitment registry); and participant characteristics and beliefs (e.g., demographics, health literacy level, and trust in government and the scientific community).

**Results:**

The focus groups and survey revealed similar findings. Participants commonly use mobile devices to go online and perform health-related activities. They were aware of AD, expressed concerns with developing it, and were willing to participate in AD-related studies (motivated by personal connection to AD, altruism, and compensation). When presented with the AD recruitment registry, most provided positive feedback (e.g., easy to use and informative) and shared an interest in joining. Barriers to joining the registry with a mobile device included complex or multistep enrollment processes, beliefs that studies are primarily for those with a specific disease, and confusion about how studies can prevent AD among those low-risk for AD. The focus groups also revealed that Black/African American participants expressed more hesitation than Hispanic/Latinos in joining the registry due to greater distrust in the government and scientific community.

**Conclusions:**

Recruiting more Black/African American and Hispanic/Latino participants into AD studies is vitally important. This mixed methods study suggests that adults in these underrepresented groups are motivated to prevent AD and willing to sign up for an AD participant recruitment registry using mobile devices. Most barriers to joining a registry can be addressed through slight modifications to the registry’s design and functionality and by adding content. These findings can help enhance the appeal of joining AD recruitment registries to ultimately enroll more diverse, representative groups of participants and increase the generalizability of AD study findings.

## Introduction

**O**ver 6 million people in the United States (US) are living with Alzheimer’s disease (AD), a number projected to increase to nearly 14 million by 2060 barring a medical breakthrough (1). Black/African American and Hispanic/Latino adults are approximately 1.5 to 2 times more likely than White, non-Hispanic/Latino adults to have AD or a related dementia (2, 3). Despite being at greater risk for developing AD, research studies often fail to enroll a diverse and representative group of participants, thereby limiting generalizability of the findings (4–6).

Online participant recruitment registries are one potential solution in helping to enroll racially and ethnically diverse participants. In the US, there are several online AD-focused participant recruitment registries, which stress the need for healthy older adults to enroll given the increasing emphasis in the field on identifying the earliest biological and cognitive changes of the disease with the goal of preventing it entirely. Unfortunately, at present, most enrollees in these registries are White, non-Hispanic/Latino women (7–9). If registries are going to play a meaningful role in helping recruit eligible participants for AD prevention and early intervention studies, it is imperative that they too mirror the larger, at-risk population.

The Alzheimer’s Prevention Registry (APR; 7) was launched in 2012, based on experience leading the Arizona Alzheimer’s Registry (10) and inspired by other large, online participant recruitment registries in other disease areas (e.g., Army of Women for breast cancer research). APR was developed using industry best practices, including considerations for health literacy, and optimized for use on a desktop computer. Since this time, the percentage of US adults who own a smartphone has increased substantially, including in adults ages 65 and older (11) and adults who identify as Black/African American or Hispanic/Latino (12). The rise in use of mobile technology presents an opportunity to revisit the design of APR.

The present study describes the results from a series of focus groups and an online survey of Black/African American and Hispanic/Latino adults ages 45–75, including those with lower health literacy, to understand the facilitators and barriers for underrepresented groups to joining a mobile-friendly AD participant recruitment registry.

## Methods

We used focus groups (n = 39) and an online survey (n = 1010) to examine barriers and facilitators. Focus groups allowed us to delve into participants’ thoughts and attitudes (13) toward APR and topics that could impact their joining APR. The survey provided a larger, more representative sample to quantitatively explore APR feedback and barriers and facilitators. Focus groups and survey participants had to be 45–75 years old; identify as Black/African American or Hispanic/Latino; own a smartphone or tablet and use them for apps or to go online at least weekly; and self-report not having a diagnosis of cognitive impairment, such as MCI or dementia due to AD. Survey participants could also identify as non-Hispanic/Latino White, a segment recruited for response comparisons. This study was approved by the Western Institutional Review Board and conducted in compliance with Good Clinical Practice guidelines, ethical regulations, and applicable local laws and regulatory requirements.

### Focus Groups

We conducted 4 focus groups in February 2020 segmented by race/ethnicity and age (45–59 and 60–75 years old). Groups with Black/African American participants (n = 9 / n = 10) were held in New Orleans, Louisiana, and groups with Hispanic/Latino participants (n = 10 for both) were held in Los Angeles, California. Regional professional focus group facilities recruited and consented participants who have previously agreed to be part of the facilities’ research panels. Trained Black/African American and Hispanic/Latino moderators conducted the groups using a structured discussion guide. All groups were conducted in English and lasted approximately 90 minutes.

The discussion guide explored current use of smartphones or tablets for health; attitudes toward and participation in health studies, including AD prevention studies; AD knowledge (i.e., risk factors, prevention methods, symptoms, and treatment options) and concerns, for self or family members; and trust in government and the scientific community. Moderators read a description of a participant recruitment registry, answered participant questions about recruitment registries, and then displayed APR on a large television screen. Participants first provided written responses to questionnaire items asking about first impressions of APR, relatability (i.e., “meant for someone like you?”), and sign-up likelihood. Afterwards, they openly shared thoughts about APR and barriers and facilitators to signing up online.

### Survey

In March 2020, we worked with a survey panel company to recruit for and distribute a nationwide online survey (n = 1010). Panel participants were recruited from a sample of over 6 million US adults via email and provided electronic informed consent prior to taking the survey. Participants were randomly emailed from within each of the three race/ethnicity segments of interest (Black/African American, Hispanic/Latino, and White) until the quota for each segment was reached. Thirty-eight percent of those who clicked on the survey and began taking it were eligible and completed it. The survey was in English and took on average 15 minutes to complete. Participants received an incentive through the survey panel company.

Like the focus group discussion guide, the survey instrument explored participants’ reactions to APR and barriers and facilitators to joining via mobile devices (e.g., use of devices for health; concerns using devices to sign up for participant recruitment registries; participation in AD-related studies; and AD knowledge and concerns). Demographic information and health literacy levels were captured. Prior to seeing APR, survey participants were provided the same recruitment registry description as focus group participants. They viewed a screenshot of the entire APR website on one web page, with web links disabled to avoid distractions.

### Measures

Use of mobile devices. Participants were asked to select all the devices they own (smartphone, tablet, laptop, desktop, or other), the device they use most often to go online, and the frequency they use mobile devices to go on apps or online (1=more than 10 times/day, 5=1–6 times/week). Participants were also asked whether they have used mobile devices for specific health-related activities in the past three months (e.g., searched information about a health condition, read an article about a health issue, and signed into electronic health records).

AD-related awareness and concern. Participants selected their level of awareness about AD or other causes of dementia (1=completely unaware, 5=completely aware) and concern about developing these conditions (1=very unconcerned, 5=very concerned). Awareness and concern were each measured using one question. Participants also shared whether they know close others (e.g., parent, sibling, spouse/partner, or friend) affected by AD or other causes of dementia.

Participation in health research. Participants were asked in one question whether they had ever participated in a health study. They were then asked how likely (1=very unlikely, 5=very likely) they would be to participate in 4 different types of AD studies: questionnaires/surveys, focus groups/interviews, brain imaging tests, and testing new pharmaceutical drugs. Responses to these 4 items were included in a principal component analysis, loaded onto a single component, and were averaged into a composite score with high reliability (M=3.38, SD=.99; α=.808).

Privacy and trust concerns signing up on mobile devices. After viewing APR, participants shared how influential (1=not at all influential, 4=very influential) the following would be in their decision to sign up for a recruitment registry using a mobile device: privacy or confidentiality concerns, concerns the organization would use their information for other purposes, and trustworthiness of the sponsor organizations. These items were included in a principal component analysis, loaded onto a single component, and were averaged into a composite score with high reliability (M=2.72, SD=1.02; α=.884).

APR feedback and likelihood of signing up on a mobile device. Participants were asked the following agreement statements (1=strongly disagree, 5=strongly agree) after viewing APR: “Is appealing,” “seems easy to use,” and “seems like it was designed with people like me in mind.” They also shared their likelihood (1=very unlikely, 5=very likely) of signing up for APR using a mobile device and were asked, in an open-ended question, to explain their response.

Health literacy level. This was determined by combined responses to three items adapted from the validated 4-item Brief Health Literacy Screening Tool (14): 1) how participants fill out medical forms (by themselves without asking questions; mostly themselves but sometimes ask questions; often ask for help; or do not feel comfortable at all filling them out without help); 2) how often someone helps participants read hospital or medical materials (1=all of the time, 5=none of the time); and 3) how often participants have problems learning about medical conditions due to difficulty understanding written information (1=all of the time, 5=none of the time). Participants were categorized as lower health literacy if they often or always ask for help filling out medical forms or if they selected 1 (all of the time) or 2 (most of the time) for the other 2 items.

Race/ethnicity and other demographics. Participants could select all race/ethnicity categories that apply to them but had to select “Black or African American,” “Hispanic or Latino,” or “White” to take the survey. A quota was imposed to ensure equal distributions for these three categories. Nine participants selected both Black/African American and Hispanic/Latino; they were categorized as Black/African American. We compared these nine participants’ responses against those from Black/African American and Hispanic/Latino participants and observed no substantial differences qualitatively when comparing responses among these groups.

Participants also wrote in their age and selected responses from lists of categories to best describe their gender, highest education level completed, and household income level.

### Data Analyses

One of the authors (DNC), experienced in qualitative data analysis, summarized focus group transcripts in detail and compared responses across the 4 groups for similarities and differences. This author also analyzed qualitative data from open-ended survey responses. Using an inductive, iterative approach, the author reviewed participant responses, developed a list of codes, and assigned codes to each response.

SPSS version 27 was used to analyze quantitative survey data. We used Chi-square tests to compare categorical responses between Hispanic/Latino, Black/ African American, and White participants, and one-way analysis of variance tests to compare continuous-level responses between these groups of participants. We used multilevel multiple linear regression (n = 907) to examine top predictors of likelihood to sign up for APR using a mobile device – level 1 examines items related to health and AD; level 2 incorporates items related to participant technology use and receptivity to APR; and level 3 incorporates participant characteristics as predictors. For all analyses, we set significant p-values ≤ 0.05 using two-tailed analyses and checked for multicollinearity where appropriate.

## Results

### Focus Groups

Responses were similar across groups in most topic areas, outlined below. The topics are not a listing of themes and also reflect topics asked in the survey. There were differences in responses related to trust in government and the scientific community between Black/African American and Hispanic/Latino participants. There were no differences in responses by age group. See Table 1 for participant demographics and other characteristics and Table 2 for sample quotes.

**Table 1:** Participant characteristics and bivariate analyses for survey responses, by race/ethnicity

	**Focus groups**		**Online survey**				
	**Black/AA**	**H/Latino**	**Total**	**White**	**Black/AA**	**H/Latino**	**Bivariate analyses for survey responses**
**N or n**	n=19*	**n=20**	**N=1010**	**n=330**	**n=341**	**n=339**	
Age (years)							F(2,1006)=14.144, p=.000
45–59	9 (47.4)	10 (50)	---	---	---	---	
60–75	10 (52.6)	10 (50)	---	---	---	---	
Continuous	---	---	60.6 (8.0)	62.1 (7.9)	60.7 (8.0)	58.9 (7.8)	
Gender†							X^2^(2)=22.383, p=.000
Male	10 (52.6)	10 (50)	393 (39.2)	96 (29.2)	141 (41.7)	156 (46.6)	
Female	9 (47.4)	10 (50)	609 (60.8)	233 (70.8)	197 (58.3)	179 (53.4)	
Education level‡							X^2^(6)=15.753, p=.015
High school or less	8 (44.4)	10 (50)	176 (17.5)	51 (15.5)	65 (19.1)	60 (17.8)	
Some college, trade school	2(11.1)	7 (35)	273 (27.1)	79 (23.9)	107 (31.5)	87 (25.7)	
College graduate	2(11.1)	3 (15)	311 (30.9)	99 (30.0)	106 (31.2)	106 (31.4)	
Graduate work, degree	6 (33.3)	0 (0)	248 (24.6)	101 (30.6)	62 (18.2)	85 (25.1)	
Household income‡							X^2^(6)=53.889, p=.000
$0–$29,999	9 (50)	6 (30)	230 (24.0)	57 (18.8)	111 (34.3)	62 (18.7)	
$30,000–$59,999	5 (27.9)	7 (35)	245 (25.5)	62 (20.4)	94 (29.0)	89 (26.8)	
$60,000–$99,999	4 (22.2)	7 (35)	236 (24.6)	76 (25.0)	63 (19.4)	97 (29.2)	
$100,000 or more	0 (0)	0 (0)	249 (25.9)	109 (35.9)	56 (17.3)	84 (25.3)	
Health literacy level							X^2^(2)=16.395, p=.000
Lower	5 (27.8)	5 (25)	89 (8.8)	13 (3.9)	33 (9.7)	43 (12.7)	
Higher	13 (72.2)	15 (75)	921 (91.2)	317 (96.1)	308 (90.3)	296 (87.3)	
Uses mobile devices most to go online	---	---	507 (50.2)	138 (41.8)	189 (55.4)	180 (53.1)	X^2^(2)=14.136, p=.001
Mobile device use frequency							X^2^(8)=5.925, p=.656
10+ times/day	10 (55.6)	5 (25)	393 (38.9)	114 (34.5)	137 (40.2)	142 (41.9)	
5–9 times/day	6 (33.3)	10 (50)	248 (24.6)	84 (25.5)	81 (23.8)	83 (24.5)	
2–4 times/day	0 (0)	4 (20)	256 (25.3)	91 (27.6)	89 (26.1)	76 (22.4)	
1 time/day	1 (5.6)	0 (0)	38 (3.8)	15 (4.5)	10 (2.9)	13 (3.8)	
1–6 times/week or less	1 (5.6)	1 (5)	75 (7.4)	26 (7.9)	24 (7.0)	25 (7.4)	
Health and AD (5-point scales, unless otherwise noted)							
Awareness of AD/other causes of dementia	---	---	3.92 (.97)	4.05 (.79)	3.87 (1.1)	3.85 (1.01)	F(2,1007)=4.407, p=.012
Concerned with developing AD/other causes of dementia	---	---	3.59 (1.05)	3.64 (.94)	3.47 (1.16)	3.66 (1.02)	F(2,1007)=3.471, p=.031
Knows close other affected by AD/other causes of dementia§	---	---	581 (60.2)	198 (62.3)	186 (58.1)	197 (60.2)	X^2^(2)=1.141, p=.565
Has participated in a health study§	---	---	250 (24.8)	90 (27.3)	93 (27.3)	67 (19.8)	X^2^(2)=6.817, p=.033
Likely to participate in AD studies	---	---	3.38 (.99)	3.29 (.95)	3.39 (1.02)	3.46 (.99)	F(2,1007)=2.343, p=.091
Technology use and receptivity to APR (5-point scales, unless otherwise noted)							
Privacy/trust concerns with registry sign-up on mobile devices∥	---	---	2.72 (1.02)	2.73 (1.02)	2.70 (1.06)	2.73 (.97)	F(2,1007)=0.148, p=.862
APR seems easy to use	---	---	3.99 (.92)	3.89 (.96)	4.04 (.89)	4.02 (.92)	F(2,1007)=2.594, p=.075
APR is appealing	---	---	3.57 (1.07)	3.48 (1.05)	3.59 (1.08)	3.64 (1.06)	F(2,1007)=1.954, p=.142
APR was designed with people like me in mind	---	---	3.49 (1.04)	3.44 (1.02)	3.49 (1.06)	3.53 (1.04)	F(2,1007)=0.608, p=.545
Likely to sign up for APR using a mobile device	---	---	3.04 (1.33)	2.86 (1.30)	3.15 (1.35)	3.09 (1.33)	F(2,1007)=4.289, p=.014

n (%N) or M (SD); AA, Black/African American; H, Hispanic; AD, Alzheimer’s disease; APR, Alzheimer’s Prevention Registry; *Some information missing for one participant; †“Another gender” category (i.e., trans male/trans man, gender queer or gender non-conforming, and something else) not displayed due to low sample size (n=7); ‡Survey sample size is smaller as some participants selected “decline” or “don’t know” response options; §Response options are “yes” or “no”; ∥Average is on a 4-point scale.

**Table 2:** Focus groups sample quotes, by topic area

**Topic area**	**Sample quotes**
Use of mobile devices for health	[I get on] Yelp to find out if it’s a legit agency or healthcare facility… to read some of the reviews. … Or going into a website like YouTube to do exercises. – Group 3
	I don’t like [when websites ask for] that much information. They get personal. … Like if you’re married, or do you vote? – Group 2
	I don’t like… registering with sites that ask lengthy questions. Now, like this brother said, putting email and information [that] pops up, and you can register, great. It takes a minute or two… something that takes longer than that, I tend not to. – Group 1
AD-related knowledge and concern	When do you [get diagnosed for AD]? Most people probably forget things already. … I’m always thinking about it because, come on, I’m getting older. – Group 4
	My dad died of Alzheimer’s. I didn’t plan on coming [to this focus group] for that reason. … The symptoms [were] lack of memory, recognition, personal items, past history, even recent history. Then it gets progressively [worse]. – Group 3
Participation in health and AD research studies	If you can go to [a study] that’s going to say, ‘Okay, this is how you prevent it,’ you would go, out of concern for yourself and for family members. – Group 1
	Someone [who participates in a health study] has an illness and is desperate. … When you bring yourself into a test, you’re like a guinea pig… it is risky. – Group 4
	[Tell] me something more than just ‘participating.’ Is there something I have to ingest? A shot? ‘Cause there’s nothing wrong with me presently. – Group 2
Trust in government and the scientific community	I don’t think they should do a study with just Black people because we are going to be fearful of, ‘What are they giving us?’ … ‘What are they trying to do?’ – Group 1
	If we had more Black scientists doing these studies, we would be interested in them. … It’s kind of a hard just to listen to somebody [say], ‘Come on, try this… This [is] gonna get you well,’ when the whole history of us believing and getting killed. – Group 2
	If that information goes to like, when you want to get a job… [an employer could find out] ‘This person’s going to get Alzheimer’s,’ so she’s [going to] have to get an outside person. That’s where you think, ‘Would the government get into that?’ – Group 4
APR testing and feedback	It has a three-step easy sign-up process. … It’s voluntary and no phone call. – Group 1
	It seems pretty easy. … Not a lot of medical terminology. It’s to the point. – Group 4
	Sponsors who are well respected in the medical community… The Mayo Clinic, Johns Hopkins… we know them from having conducted legitimate studies. – Group 2
	What are you preventing? If you don’t have a family member or don’t know anybody that had Alzheimer’s, you’re not going to know what you’re going to prevent. – Group 4
	I’m skeptical of anything that comes over my phone. I would have to be told by my physician or a person that’s close to me. – Group 1

AD, Alzheimer’s disease; APR, Alzheimer’s Prevention Registry; Group 1: Black/African American, 60–75 years old; Group 2: Black/African American, 45–59 years old; Group 3: Hispanic/Latino, 60–75 years old; Group 4: Hispanic/Latino, 45–59 years old.

### Use of mobile devices for health

Participants shared that they routinely use smartphones for health information, via Google searches, websites (e.g., WebMD), or social media (e.g., Facebook, Yelp, or YouTube). Several mentioned that smartphones are convenient for checking information (e.g., medication side effects or healthcare facility ratings) when needed or desired. When signing up for accounts online, participants preferred quick registration processes and few requirements (e.g., only an email address and password). Websites that ask more personal questions or include more stringent security measures (e.g., security questions, complex passwords, and CAPTCHA tests) were perceived as inconvenient and, along with multi-item registrations, were barriers to signing up for some participants.

### AD-related knowledge and concerns

Some participants across groups knew family members or close others with AD, which some called “scary,” “worse than cancer,” and “a horrible death.” Several mentioned that AD can be hereditary and offered suggestions for prevention (e.g., puzzles and better nutrition); however, none believed there is a cure. Several shared concerns about developing AD and many wished to learn more about AD prevention or how to slow its progression.

### Participation in health- and AD-related studies

Overall, participants believed that health studies were mainly for those who are already ill or seeking help or treatments for ailments. Several shared that they have previously participated or want to participate in studies for conditions that they have (e.g., psoriasis, hypertension, and knee pains), or knowing a close other who has participated in health studies. When asked about AD studies for prevention, participants shared some level of interest; however, there was confusion and questions about the nature of prevention-based studies and apprehension, by some, if medications were involved. When asked broadly about motivations for participating in AD-related studies, several mentioned AD relevance (e.g., family member has it or had it), altruism (helping others or advancing science), and compensation.

### Trust in government and the scientific community

Participants were asked whether trust in government and in the scientific community would influence their decision to join a participant recruitment registry. Black/African American participants shared greater distrust, compared to Hispanic/Latino participants, and concerns about research studies focusing only on Blacks/African Americans and the government accessing and misusing their health information. Some attributed distrust to historic racial discrimination against Blacks/African Americans in the US. A few mentioned trusting churches and other community organizations, and Black/ African American scientists and doctors. Hispanic/Latino participants shared concerns of health information being shared without their knowledge or consent, particularly with employers. One Hispanic/Latino participant shared that trust in government depends on types of health agencies (e.g., national vs. county) supporting a registry.

### APR testing and feedback

Most participants (n = 29) said that they would sign up or shared positive thoughts in written responses to the question, “Do you think you would sign up for this registry?” In the group discussions, participants first shared general impressions of APR and anything that stood out. Moderators asked structured follow-up questions as needed, inquiring into information or components of APR perceived as useful, engaging, confusing, or relatable; perceived trustworthiness; and general likes and dislikes.

Participants across groups shared a lot of positive feedback. They described APR as easy to navigate and read, and as informative yet brief. There were positive mentions of AD educational information in the “latest news” section as some were motivated to learn more about AD. Those who commented on the sign-up process described it as straight-forward and non-intrusive (e.g., non-personal information required, and voluntary participation clarified). A few also found the “why now?” section as motivational to registering as it helps describe why more participants are needed for AD recruitment registries. Many described APR as trustworthy; some commented favorably on the display of number of registrants and on the list of sponsor/ partner organizations, which participants recognized and considered medically reputable.

Most of the negative feedback of APR was related to the word “prevention” as it was unclear how studies could prevent AD, particularly if participants did not have a family history of AD or perceptions of risk. A few others shared general distrust signing up for something health-related without recommendations from a medical provider.

### Survey

A total of 1010 participants completed the survey. (The sample size was based on an a priori power of 80% (comparing the three race/ethnicity groups on likelihood of signing up for APR on a mobile device) and type I error of 0.05.) Nearly two-thirds (63.5%) use mobile devices at least five times daily to go on apps or online (Table 1). All participants performed at least one health-related activity on a mobile device in the past three months, and 72.5% performed two or more (not displayed) – searching for information about a health condition (67.5%) and reading articles about a health issue (56.6%) were most common. There were significant differences in participant characteristics and use of mobile devices by race/ ethnicity (Table 1). White participants were more likely than participants in the other groups to be female and to report higher levels of education, household income, and health literacy.

Per Table 1, most (60.2%) reported knowing close others affected by AD or other causes of dementia. White participants reported greater AD awareness (F(2, 1007) = 4.407, p = .012, η2 = .009) and Black/African American participants were least concerned about developing AD (F(2, 1007) = 3.471, p = .031, η2 = .007). A quarter of participants (24.8%) have participated in a health research study, with Hispanic/Latino participants (19.8%) less likely to have done so (X^2^(2) = 6.817, p = .033, V = .082). However, participants across race/ethnicity categories reported similar likelihood of participating in AD studies (total: M = 3.38, SD = .99).

Participants across categories also reported similar receptivity to APR and privacy/trust concerns signing up for registries on mobile devices. However, White participants were less likely to sign up for APR on a mobile device compared to others (F(2, 1007) = 4.289, p = .014, η2 = .008). Open-ended response themes indicated that participants’ likelihood to sign up was related to feelings of (dis)comfort or privacy concerns using mobile devices for this activity, receptivity to APR, and beliefs that AD is personally relevant (Table 3). Those likely to sign up also shared positive thoughts about participating in research studies.

**Table 3:** Themes of open-ended survey responses to, “How likely would you be to sign up for [APR] using your smartphone or tablet?”

**Sign up likelihood**	**Prominent themes**	**Sub-themes, if applicable**
Very unlikely or unlikely (n=311)	Not comfortable using mobile devices to sign up	Prefers not to sign up via smartphone/tablet
		Prefers desktop or laptop
		Has trouble reading/typing on mobile devices
	Has privacy/security concerns	---
	AD is not relevant to participant	---
	Negative feedback about APR	Provides too much information
		Is not trustworthy/reputable
	Generally disinterested	---
Neutral (n=228)	Lacks time to participate	---
	Generally undecided	Would like more information
Very likely or likely (n=353)	Comfortable using mobile devices to sign up	Prefers using smartphone or tablet
		Mobile devices are most convenient
		It is easy to sign up on a mobile device
	AD is relevant to participant	---
	Positive thoughts on participating in research studies	Generally open to participating in research
		Signing up can be educational
		Altruism (to help science, the cause, other people)
	Positive feedback about APR	Easy to read, navigate, or use
		Is trustworthy/reputable
		Is appealing
		Is informative
	Generally interested	---

AD, Alzheimer’s disease; APR, Alzheimer’s Prevention Registry.

A multilevel multiple linear regression model reveals several significant predictors of likelihood to sign up for APR using a mobile device (Table 4). Predictors related to technology use behaviors and beliefs, including APR receptivity (level 2), greatly increased the model’s predictive power (R^2^ change = .258). In the full model, likelihood of participating in AD studies was the only significant predictor in level 1 (predictors related to health and AD) and, among participant characteristics (level 3), lower income and lower health literacy levels were positively related to the outcome. Education level was nearing statistical significance. The model is a strong fit for the data (adjusted R^2^ = .495).

**Table 4:** Predictors of likelihood to sign up for APR using a mobile device (n = 907)

**Predictor**	**Level 1**		**Level 2**		**Level 3**	
	**Health and AD**		**Use of mobile devices and APR receptivity**		**Participant characteristics**	
	Beta*	**p-value**	Beta*	**p-value**	Beta*	**p-value**
Awareness about AD or other causes of dementia	.016	.600	−.027	.274	−.024	.325
Concerned about developing AD or other causes of dementia	.115	.000	.035	.170	.036	.158
Knows close other affected by AD or other causes of dementia†	−.017	.567	−.035	.151	−.030	.224
Has participated in a health research study†	−.046	.112	−.046	.053	−.038	.112
Likely to participate in AD research studies	.444	.000	.229	.000	.221	.000
Uses smartphone or tablet most to go online†	---	---	.199	.000	.191	.000
Smartphone or tablet use frequency	---	---	.102	.000	.097	.000
Privacy/trust concerns with registry sign-up on mobile devices	---	---	.052	.034	.051	.039
APR seems easy to use	---	---	.044	.153	.050	.106
APR is appealing to me	---	---	.313	.000	.304	.000
APR was designed with people like me in mind	---	---	.149	.000	.153	.000
Race/ethnicity‡	---	---	---	---	−.005	.844
Age (continuous)	---	---	---	---	−.026	.296
Gender§	---	---	---	---	−.041	.109
Education level	---	---	---	---	.051	.056
Income level	---	---	---	---	−.063	.021
Health literacy level∥	---	---	---	---	−.059	.015
Model details						
R^2^	.237		.495		.504	
Adjusted R^2^	.233		.489		.495	
R^2^ Change	.237		.258		.009	

AD, Alzheimer’s disease; APR, Alzheimer’s Prevention Registry; *Standardized coefficient; †0=no, 1=yes; =‡=0=White, 1=Black/African American or Hispanic/Latino; §0=Male, 1=Female; 110=Lower health literacy, 1=higher health literacy.

## Discussion

To date, AD-focused online participant recruitment registries have not been successful at enrolling participants from underrepresented communities. In the present study, we conducted a series of focus groups and a nationwide online survey to examine barriers and facilitators for cognitively unimpaired adults who identify as Black/African American or Hispanic/Latino, including those with lower health literacy, to joining APR. This study focused on mobile devices (i.e., smartphones and tablets) given the rise in use of mobile technology among older adults, including those who identify as Black/African American and Hispanic/Latino.

The focus groups and survey revealed similar findings. Differences among racial and ethnic groups were only evident on select questions. When focus group participants were asked broadly about motivations for participating in AD-related studies, three main themes emerged, consistent with previous studies (15–18): personal connection to the disease, altruism and advancing science, and compensation. In general, focus group participants routinely use smartphones when searching for health information. This mirrors results from the online survey, which found that Black/African American and Hispanic/Latino respondents use mobile devices to go online more than White respondents. Among focus groups participants, barriers for using a mobile device for joining a registry include complex or multistep enrollment processes (e.g., security questions, complex passwords, CAPTCHA tests). Participants were generally aware of AD and expressed concern for developing the disease. This mirrors the results from the online survey, though the survey found that White participants were more aware about AD in general and Black/African American participants were less concerned about developing AD.

Focus group participants considered health-related studies to be primarily for people with a specific disease or condition; participants were generally unaware of AD prevention studies and expressed apprehension about studies involving medications. Concerns about medical experimentation are consistent with prior studies (15, 19, 20). The only difference between Black/African American and Hispanic/Latino adults emerged when focus group participants were asked whether trust in government and the scientific community would influence their decision to join a registry, a theme commonly reported in prior studies (21–23). Although participants from both racial/ ethnic groups expressed some distrust, Black/African American participants expressed greater distrust and also shared concerns about studies focused only on Black/ African Americans. Although general distrust was not measured in the survey, we assessed privacy/trust with joining a registry on a mobile device and did not find a significant difference between racial/ethnic groups.

Health literacy is an important factor to consider for study recruitment of older adults from underrepresented populations. US adults who are 65 years or older and part of racial and ethnic minorities are more likely to report lower health literacy (24). In this study, lower health literacy was associated with greater likelihood to sign up for an AD participant recruitment registry using a mobile device, after controlling for other predictors. Prior studies assessing health study recruitment and participation and health literacy levels among older, racially/ethnically diverse participants have reported different findings; specifically, health literacy levels are unrelated to recruitment and retention (25) and higher health literacy levels are related to study interest and participation (26). More research is needed to better understand associations between health literacy levels and health study recruitment in underrepresented populations. However, findings from this study suggest that evidence-supported and tailored approaches to designing mobile-friendly recruitment registries can play a role in captivating those who report lower health literacy.

The present study found that most participants said they would join, or shared positive feedback about APR. Importantly, most barriers to joining are largely addressable or modifiable. For example, preventing AD through studies was unclear to a large number of focus group participants, particularly if they did not have a family history of AD or did not perceive themselves to be at risk. A smaller number of focus group participants shared that they were distrustful of health-related websites that were not recommended by their healthcare provider. Both barriers are addressable through modifications to the design and functionality of APR, as well as additional content on the registry itself that could be bolstered by larger, national education campaigns to the public and medical providers (27–29). Similarly, concerns about mistrust can be addressed partially by clearly indicating the organizations or researchers leading the registry, as well as the registry’s privacy, confidentiality, and security policies. Several participants in this study commented on the sponsor organizations and researchers listed in APR and whether they trusted them, and some mentioned wanting more information on how their information would be kept safe. To make the registry accessible to those with lower health literacy, it is important to use graphics and clear, concise language (not long prose) to convey all information.

### Limitations

There are some limitations to the present study. First, there was very little negative feedback about APR during the focus groups. This may be due to a social desirability bias. It remains unknown whether these same individuals would join APR on their own. Second, the online survey was not a representative national sample. Respondents were part of an existing panel and recruited randomly from the survey company’s panel of over 6 million adults to ensure nearly equal numbers of White, Black/ African American, and Hispanic/Latino participants. Study panels provide a cost-effective way to reach study participants; however, they are subject to selection bias. For example, in this study, approximately half of survey participants were college graduates (although this varied slightly by race/ethnic group), and household income varied significantly between groups, suggesting we did not reach as many individuals of lower socioeconomic status as planned. When looking at predictors of likelihood to sign up for APR, we controlled for these factors. Further, due to time limitations on the survey, we did not capture other measures of social determinants of health, and many constructs were captured using single-item questions. However, questions that were combined to create a composite score were statistically checked for validity.

Regarding the focus groups, they were hosted in only two geographic areas (New Orleans and Los Angeles), which could be a possible confounder. These sites were chosen to provide some geographic diversity and access to Black/African American and Hispanic/Latino participants. While additional sites may have benefitted the study, we were only able to conduct four groups in two areas due to a limited budget.

Lastly, our work focused on two traditionally underrepresented groups in AD-focused participant registries: Black/African American and Hispanic/Latino adults. As a result, our findings may not generalize to other underrepresented racial and ethnic groups or cultures. Future efforts will focus on other minorized groups. In addition, future efforts should explore barriers and facilitators within racial/ethnic groups. Related, our work did include individuals with lower health literacy but was focused on individuals who can read English. Other registries, such as the CARE Registry (30), have been designed with the specific cultural and linguistic needs of the particular target audience. Because APR is currently available in English only, we did not conduct any research in Spanish. Future plans include developing a version in Spanish. This would require conducting formative research in Spanish, as simply translating the registry content would be unlikely to result in a culturally-competent program.

Despite these limitations, the results from the focus groups and survey were generally consistent with each another and with prior studies. The results from the present study suggest that APR is appealing to diverse audiences and many of the barriers identified are addressable through modifications of the information/ content on the website and technology functionality. Next steps include iteratively testing different prototypes of the mobile-friendly registry to address the facilitators and barriers identified to increase enrollment of traditionally underrepresented populations.

## Notes

*Ethical standards:* This study was approved by the Western Institutional Review Board and conducted in compliance with Good Clinical Practice guidelines, ethical regulations, and applicable local laws and regulatory requirements.
